# Datasets of a novel bivalent single chain antibody constructed by overlapping oligonucleotide annealing method targeting human CD123

**DOI:** 10.1016/j.dib.2016.07.014

**Published:** 2016-07-15

**Authors:** Shima Moradi-Kalbolandi, Mahdi Habibi-Anbouhi, Majid Golkar, Mahdi Behdani, Gashin Rezaei, Leila Ghazizadeh, Mohsen Abolhassani, Mohammad Ali Shokrgozar

**Affiliations:** a National Cell Bank of Iran, Pasteur Institute of Iran, Tehran, Iran; b Parasitology Department, Pasteur Institute of Iran, Tehran, Iran; c Biotechnology Research Center, Venom & Bio therapeutics Molecules Lab, Pasteur Institute of Iran, Tehran, Iran; d Immunology Department, Hybridoma Lab, Pasteur Institute of Iran, Tehran, Iran

## Abstract

Current therapies for acute myeloid leukemia (AML), are associated with high relapse rates. Hence, development of new therapeutic strategies is crucial to circumvent this problem. Bivalent antibody technology has been used to engineer novel antibody fragments with increased avidity, by assembling two scFv in a single molecule. Here, we present accompanying data from construction and characterization experiments of a biscFv antibody targeting CD123, the most important biomarker of leukemic cancer stem cells which play a key role in relapsed AML after chemotherapy. Data in this article are related to the research paper “Development of a novel engineered antibody targeting human CD123” Moradi-Kalbolandi S. et al. (2016) [1].

**Specifications Table**

**Table:** 

Subject area	*Molecular Biology, Biotechnology*
More specific subject area	*Antibody Engineering*
Type of data	*Table, figure*
How data was acquired	*PCR, Sodium dodecyl sulfate polyacrylamide gel electrophoresis (SDS-PAGE)*,*Westernblotting, ELISA, Flow cytometry*
Data format	*Raw, analyzed*
Experimental factors	*Assembly and characterization of the anti-CD123 biscFv construct in pET22-b expression vector and Periplasmic protein expression, purification and characterization of biscFv*
Experimental features	*Molecular Biology methodologies(PCR, enzyme restriction, DNA ligation, Protein expression), flow cytometry, ELISA*
Data source location	*Tehran, Iran*
Data accessibility	*Data is within this article*

**Value of the data**

• A detailed process for construction of an anti CD123 biscFv through scFv dimerization is described.
• The data include an experimental guide for construction of flexible linker (G_4_S)_3_ by annealing of 2 overlapping oligonucleotides without any digestion needed for cloning purpose.
• Protein expression and purification data show the expression and proper protein purity of constructed biscFv by Immobilized Metal Affinity chromatography (IMAC).
• The methods and data describe the improvement of the biscFv functions over its parental scFv.

## 1 Data

**Fig. 1:**
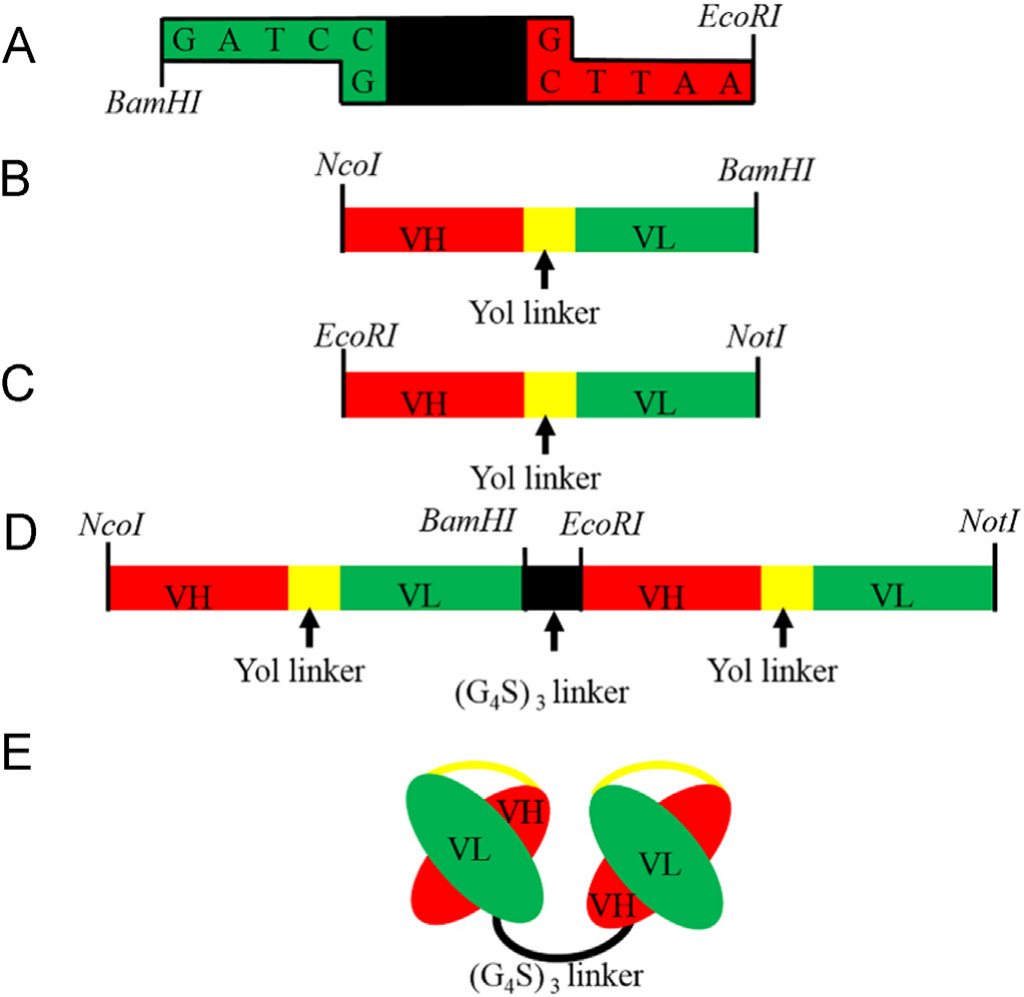
Schematic generation of biscFv. A) dsDNA coding sequence for (Gly_4_Ser)_3_ linker results from annealing of synthesized overlapping oligonucleotides L-1 and L-2, after heating for 10 min at 94 °C and cooling slowly to room temperature. Overhangs of *BamHI* and *EcoRI* were formed after annealing for cloning purpose without digestion requirement. B) scFv-1, by PCR using primer scFv1-F, scFv2-R with *NcoI, BamHI* restriction sites applied. C) scFv-2, by PCR using primer scFv2-F, scFv2-R with *EcoRI, NotI* restriction sites applied. D) BiscFv cassette, results from cloning of scFv-1, linker, scFv-2 in to pET22-b vector respectively. E) Protein form of biscFv.

**Fig. 2:**
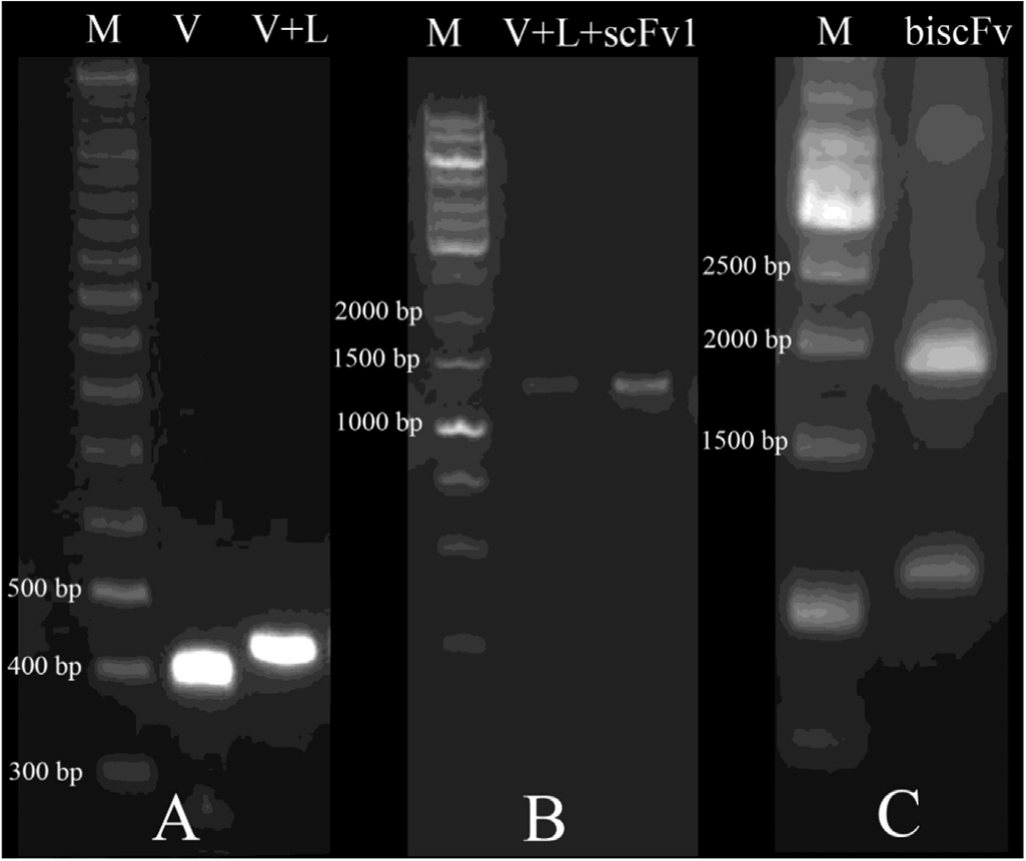
Construction of biscFv, colony PCR following insertion of each steps. A) Colony PCR following insertion of linker within the plasmid. Lane M: GenRuler DNA ladder (Thermo fisher scientific, USA), Lane V, the empty plasmid, Lane V+L ~450 bp-band, confirmed the insertion of 50 bp linker (L) in to vector (V). B) Colony PCR after second cloning procedure. The ~1250 bp-band indicates the insertion of scFv1 in to the vector contains the linker (V+L+ scFv-1). C) Colony PCR of last step cloning. The ~2000 bp-band indicates of biscFv (scFv1+L+scFv2).

**Fig. 3:**
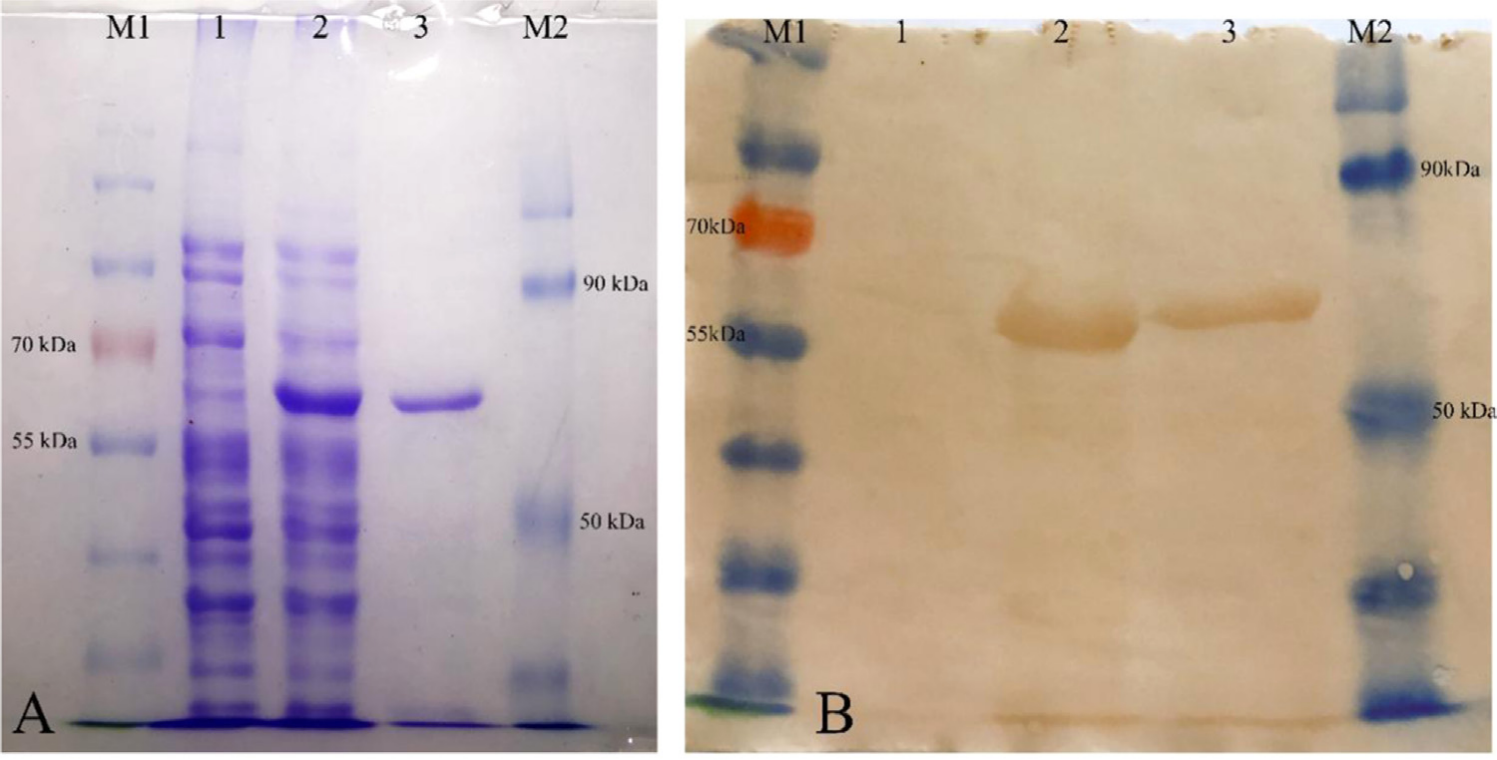
Expression and purification of biscFv. A) SDS-PAGE of expression, Coomassie brilliant blue staining. Lane M1 and M2: PageRuler prestained protein ladder (Thermo fisher scientific, USA), Lane1: periplasmic extract of *E.coli* BL21 without pET22-biscFv plasmids (negative control), Lane2: periplasmic extract before passing into the HisTrap column and lane3: purified biscFv. B) Western blot of purified biscFv using anti-His monoclonal antibody and HRP conjugated goat-anti mouse. Lane M1 and M2: PageRuler prestained protein ladder, lane 1: periplasmic extract of *E.coli* BL21 without pET22-biscFv plasmid as negative control, lane 2: periplasmic extract before passing into the HisTrap column, and lane3: purified biscFv.

**Fig. 4:**
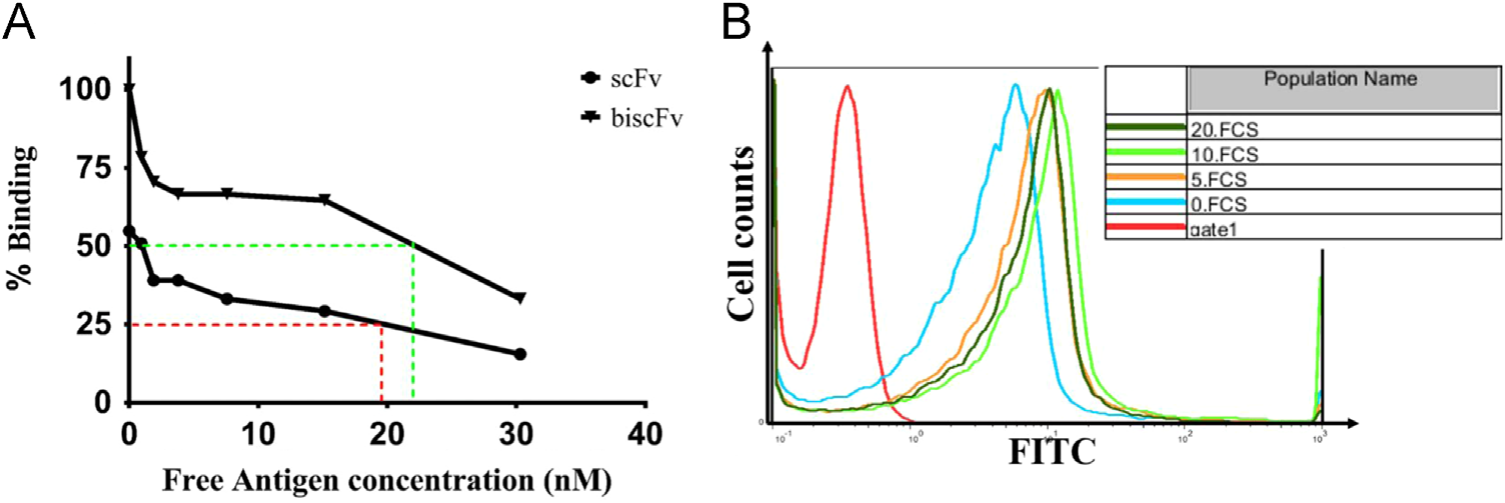
Competitive binding assays by ELISA and flow cytometry. A. Apparent (Relative) affinity determination of dimeric/monomeric scFvs formats with the competitive-ELISA method. Antibody affinity was obtained through measuring the IC_50_. These results demonstrate that the concentration required for 50% inhibition of the binding was similar for both scFv and biscFv (red and green dashes, respectively), indicating the same intrinsic affinity.But more biscFv remained bound to immobilized CD123 obtained by ELISA, indicating increase in avidity of biscFv. B) Competitive binding assay of purified biscFv and commercial anti-CD123 mAb by flow cytometry. As shown, no obvious shift in fluorescence value were observed with different concentration of biscFv (added to the cells prior to incubating commercial anti-CD123 mAb compared to background staining in the absence of biscFv.

**Fig. 5:**
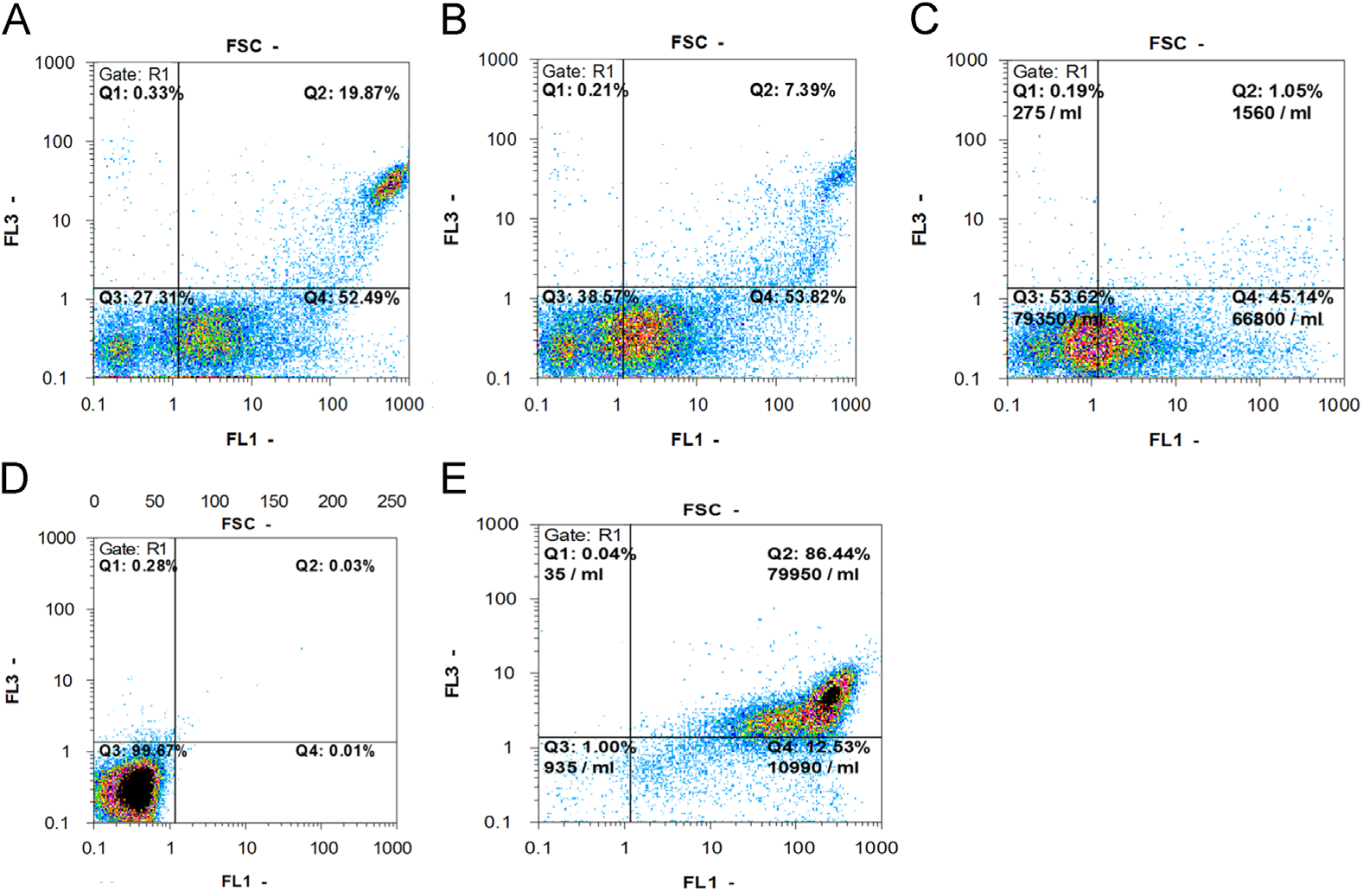
Proliferation -Apoptosis assays by flow cytometry. TF-1 cells were cultured in RPMI with 10% FBS and treated as describes bellow prior to addition of IL-3 and examined for Annexin V-FITC binding and 7-AAD uptake with flow cytometry after 24 h treatment. The percentage of cells in the lower right (LR, Annexin V^+^/7-AAD^−^) and upper right (UR, Annexin V^+^/7-ADD^+^) regions, indicate early apoptotic cells and late apoptotic cells respectively. A) Treatment with commercial anti-CD123 mAb. B) Treatment with anti-CD123 biscFv. C) Treatment with anti-CD123 scFv. D) Negative control, Treatment with PBS. E) Positive control, cells cultured in the absence of IL-3.

The cloning strategy for subsequent expression and purification of anti-CD123 biscFv were described (Fig. 1, Fig. 2 and Table 1). Expression data by SDS-PAGE and westernblotting were generated to validate the construction of anti-CD123 biscFv plasmid (Fig. 3). The bivalent scFv exhibited improved functionality compared to monovalent scFv antibody (Figs. 4A, 5 and Table 2).

## 2 Experimental design, materials and methods

### 2.1 DNA constructs

The anti-CD123- scFv plasmid, encoding the murine anti-CD123 scFv was previously isolated from our constructed scFv phage display library [2]. It is in the VH-linker-VL format, where the linker consists of 18 amino acid residue of pSEX81 phagemid (PROGEN Biotechnik GmbH, Heidelberg, Germany) (Fig. 1). Thus, scFv-110, was employed as PCR template for generation of biscFv-110, using the primers shown in Table 1. DNA amplification by PCR, digestion with restriction enzymes, ligation and other standard cloning procedures are followed well established protocols [3]. Our biscFv, was constructed by covalent linking of two individual scFv DNA sequences in tandem via a linker as shown schematically in Fig. 1. This was achieved by ligation of PCR amplified scFv1, scFv2 fragments and hybridized L fragment ((Gly_4_Ser)_3_ linker), as the building blocks, in to the pET22-b vector, separately (Fig. 2). All the above PCR fragments were amplified with *pfu* high fidelity DNA polymerase (Thermo fisher scientific, USA).

### 2.2 Expression and purification of biscFv

For soluble expression, pET22b-biscFv plasmids, were transformed into *E. coli* BL21 DE_3_ (EMD-Millipore, USA). A single recombinant colony was selected and inoculated overnight in 5 ml 2xTY containing 100 μg/ml ampicillin at 37 °C. A fresh Terrific Broth (TB) medium containing 100 μg /ml ampicillin was inoculated with the overnight culture and grown shaken in baffled flasks at 200 rpm at 37 °C until the OD600 reached 0.7–0.8. Then, expression was induced by Isopropylthio-β-galactoside (IPTG) to a final concentration of 0.3 mM, for 4 h at 26 °C. Cells were harvested by centrifugation at 3000 rpm for 30 min, and periplasmic extracts were prepared from pelleted bacteria after a cold osmotic shock. In brief, bacterial pellets were resuspended in ice-cold 1X TES buffer (0.2 M Tris–HCl pH 8.0, 0.5 mM EDTA and 0.5 M sucrose). Afterwards, cells were vortexed for 30 s and incubated on ice for 1 h, followed by resuspension in 2 volumes of TES/4 (1:4 diluted TES with dH_2_O v/v), incubated on ice for 3 h. The supernatant containing soluble periplasmic proteins was recovered by centrifugation at 3000 rpm for 30 min at 4 °C. Then the his-tagged biscFv was purified from periplasmic extract by immobilized metal affinity chromatography (IMAC) using Ni-NTA-Agarose column (Qiagen, Germany), according to the manufacturer׳s instructions.

## 3 Characterization of purified anti CD123 biscFv

### 3.1 SDS-PAGE, Western blotting

Following expression in *Ecoli.*BL21 and purification by immobilized metal affinity chromatography (IMAC), the Purified biscFvs were analyzed by SDS-PAGE and western blotting using prestained Protein Page Ruler (Thermo Fisher Scientific, USA) as described below. After electrophoresis, the protein bands were visualized by Coomassie Brilliant Blue staining or were electrophoretically transferred to a nitrocellulose membrane and blocked for 16 h at 4 °C using 3% BSA in Tris-buffered saline (TBS), followed by four washes of 5 min in 20 ml of TBS, 0.1% (v/v) Tween 20. Mouse anti-His tag mAb (Qiagen, Germany) diluted 1:3000 in TBS, was added to detect the biscFv for 1 h at RT. Following four washes, the membrane was treated with HRP conjugated anti-mouse antibody (Sigma, Germany), subsequently revealed by 3, 3-diaminobenzidine (DAB) (Sigma, Germany) after four washes as previously described (Fig. 3).

### 3.2 Antigen binding activity

Indirect ELISA was performed to evaluate the antigen-binding activity of purified anti-CD123 biscFv. A microtiter ELISA plate (Maxisorp, Nunc, Denmark) were coated with 100 µl of 1 µg/ml recombinant CD123 (R&D Systems, USA) in PBS, or PBS alone as a negative control at 4 °C overnight. After discarding the coating solution, wells were blocked with 3% BSA (w/v) for 1 h at room temperature. Wells were then washed three times with PBST (PBS with 0.05% Tween 20 v/v), followed by dispersing 100 µl purified biscFv (1 µg/ml) into the wells for 2 h at room temperature. Then wells were washed three times with PBST followed by dispersing mouse anti-His tag mAb (1:3000 v/v) (Qiagen, Germany) into the wells for 1 h at room temperature. Finally the plate was washed, incubated with HRP conjugated anti mouse antibody (Sigma, Germany) for 1 h and the peroxidase activity was detected by 3, 30, 5, 50-tetramethylbenezidine (TMB) (Sigma, Germany). The reaction was stopped by 1 M sulfuric acid and color absorbance was determined at 450 nm using an ELISA microtiter plate reader (Stat Fax 2100, Awarness Technologies, USA).

### 3.3 Competitive ELISA

To investigate bivalent avidity effects, competitive ELISA were done according to Rath et al. [4]. Briefly, recombinant CD123, was coated in a microtiter ELISA plate at a concentration of 1 μg/ml in PBS. After washing with PBS and blocking with 3% BSA in PBS, the biscFv and scFv at a concentration of 1 μg/ml in PBS, were allowed to bind to the immobilized CD123 for 1 h at 37 °C, separately. Unbound antibodies were removed by three times washing with PBST (PBS with 0.05% Tween 20 v/v). Recombinant CD123 protein at a decreasing concentrations of 30.3, 15.15, 7.58, 3.79, 1.89, 0.945 nM were applied to the wells with positive (without antigen) and negative (without biscFv-scFv)controls, for 1 h at 37 °C. At the end of the incubation time, the plate was washed and bound antibody was detected as described above. The ratio of antibody molecules remained bound to the plate in the presence of solution-phase competing CD123 to those of the wells did not contain competing CD123 was determined and depicted as the concentrations of solution-phase CD123 versus the percentage of antibody remaining bound to the coated CD123. Subsequently, the binding affinities of scFv and biscFv for CD123 were determined as IC_50_ values by GraphPad Prism 5 software (La Jolla, SanDiego, USA), defined as the concentration of free CD123 that blocked 50% of the antibody binding to the immobilized CD123 [5], [6] (Fig. 4A).

### 3.4 Competition- binding assay between commercial anti-CD123 mouse mAb and anti-CD123 biscFv by flow cytometry

To assess whether the binding epitope of our biscFv interferes with binding epitope of commercial anti-CD123 mAb (BD Pharmingen, USA), a competition-binding assay by flow cytometry was utilized. 5×10^5^ TF-1 cells were incubated with the prepared biscFv, with increasing concentration of 5, 10 and 20 µg/ml, for 1 h at 4 °C. Cells were then incubated with a constant concentration of the commercial anti-CD123 mAb (1 µg/ml) for 1 h at 4 °C. Following, three more washes cells re-incubated with goat anti-mouse FITC conjugated antibody (1:500 in PBS-FBS 4%) (Abcam, UK) for 1 h at 4 °C. Cells were then washed and analyzed on a FACS Calibur flow cytometry machine (Partec, Germany). The obtained data was analyzed using FlowJo 9.7 (Treestar, Ashland, OR) and were shown in Fig. 4B.

## Figures and Tables

**Table 1:** Primers used for PCR of scFv genes and oligonucleotide for linker in order to biscFv construction.

Oligonucleotide	5^′^ to 3^′^ sequence^a^
L1	GATCCGGTGGAGGCGGTTCAGGCGGAGGTGGCAGCGGCGGTGGCGGGTCGAG
L2	GCCACCTCCGCCAAGTCCGCCTCCACCGTCGCCGCCACCGCCCAGCTCTTAA
scFv1-F^b^	GTATGTTGTGTGGAATTGTG
scFv1-R	TAGGATCCGTTTGATTTCCACCTTG
scFv2-F	TTGAATTCTTGAGGTGCAGCTGTTGGAGTC
scFv2-R^b^	GTTTTGTCGTCTTTCCAG

a Restriction sites showed by underline.

b Restriction sites of *NcoI* and *NotI* are within the PCR products.

**Table 2:** Comparison of anti-IL-3 activity of different treatments on TF-1 cells.

Treatments	scFv	aSD	BiscFv	SD	Commercial Anti-CD123 mAb	SD	bControl+	SD	cControl−	SD
dMean % Anti-IL-3 Activity	46.48%	0.64	60.79%	0.70	72.4%	1.1	97.3%	0.87	0.6%	0.37

The results were analyzed with one-way ANOVA and the *P* value<0.0001.

a The standard deviation of means from triplicate in given experiment.

b Positive controls (TF-1 cells cultured in the absence of IL-3).

c Negative controls (TF-1 cells treated with PBS).

d Total mean% cells of early and late apoptosis from triplicate in given experiment.
