# Supplementary material for: Datasets of a novel bivalent single chain antibody constructed by overlapping oligonucleotide annealing method targeting human CD123
Source: Data Brief. 2016 Jul 15;8:1137–43. doi: 10.1016/j.dib.2016.07.014 (PMC4976644; doi:10.1016/j.dib.2016.07.014)
Supplement: Supplementary file 1 — Supplementary material [file mmc1.pdf]

جمهوری اسلامی ایران

Pasteur Institute of Iran

Dear Sarah O'Loughlin,
Managing Editor of Data in Brief,

We are pleased to resubmit this manuscript entitled ***"Datasets of a novel bivalent single chain antibody constructed by overlapping oligonucleotide annealing method targeting human CD123"*** with reference NO. DIB-D-16-00340. We would like to thank both reviewers and editor, for their insightful comments on the paper, as these comments led us to an improvement of the work.

This manuscript contains original data and has not been considered for publication in other journals and if accepted will not be published elsewhere in the same form, in any language, without the written consent of the publisher. All coauthors have approved the manuscript and will send a consent letter in due course. Also, there are no conflicts of interest in this work.

Yours Sincerely;

Mohammad Ali Shokrgozar

Corresponding author

69,pasteur Ave.Tehran-13164
Tel:+98 21 6953311-20
Fax:+98 21 6465132
E-mail:office@institute.pasteur.ac.ir
Web page: http://www.pasteur.ac.ir
